# Understanding occupational therapy perinatal mental health practice in mothers from ethnic minorities: A qualitative study of practitioner perceived barriers and enablers

**DOI:** 10.1177/03080226241295602

**Published:** 2024-11-04

**Authors:** Kathryn Halsall, Kath Ward, Kathryn Jarvis

**Affiliations:** 1 Lancashire and South Cumbria NHS Foundation Trust, Preston, UK; 2 School of Health, Social Work and Sport, University of Central Lancashire, Preston, UK; 3 Stroke Research Team, University of Central Lancashire, Preston, UK

**Keywords:** Occupational therapy, perinatal mental health, cultural competence, barriers, ethnic minority, qualitative

## Abstract

**Introduction:**

In the United Kingdom, ethnic minority mothers experience increased risk of mental illness compared to white women of British heritage. However, there is insufficient research to guide perinatal mental health occupational therapists to develop services that are accessible and meet the diverse needs of service users. This study explored perinatal mental health occupational therapists’ perceptions of the barriers and enablers to an inclusive service provision for ethnic minority mothers.

**Methods:**

A qualitative study was undertaken. Recruitment via social media used non-probability sampling. Semi-structured interviews collected data which were then analysed thematically.

**Results:**

Eight occupational therapist participated in the study. Three main themes were identified: observation of caseloads; experience of providing occupational therapy; influence of the therapist’s culture. Participants perceived stigma, fear, language and cultural perceptions created barriers for ethnic minority mothers.

**Discussion:**

Limited workforce diversity, ineffective mandatory training and insufficient referral to occupational therapy by other healthcare professionals were believed to negatively impact service delivery. Cultural experience and reflective practice were felt to enable inclusive practice. Recommendations to inform perinatal mental health occupational therapy practice in the United Kingdom and internationally include collaborations to gain a more diverse workforce, improved mandatory training and strategies to increase cultural sensitivity and competence.

## Introduction

The perinatal period extends from conception to the first postnatal year, during which up to 20% of women from the United Kingdom (UK) will experience mental health problems (Royal College of Midwives, 2020). The human and economic cost of perinatal mental illness is high with suicide being the leading direct cause of maternal death in the UK (MBRRACE-UK, 2023). The long-term financial impact of perinatal mental illness is estimated to cost the UK economy £8.1 billion per cohort per year (Bauer et al., 2022).

Over the last few years funding for perinatal mental health has been significantly increased (National Health Service, 2019a) with a national programme of specialist Perinatal Community Mental Health Services (PCMHS) being developed under the guidance of the Royal College of Psychiatrists. The UK is currently one of the world leaders in the development of perinatal mental health care (Horakova et al., 2024) with occupational therapy being integral to the clinical model. Perinatal mental health occupational therapists provide specialist assessment, intervention and monitoring of the mother’s occupational needs and functioning in the context of perinatal mental ill health and parenthood (Perinatal Quality Network, 2020). The current UK recommendation for staffing is a minimum of two occupational therapists per 10,000 births (Royal College of Psychiatrists, 2021) equating to approximately 120 posts in England and Wales (Office for National Statistics, 2021). Since 2024, PCMHS teams have been commissioned to provide a service up to 24 months post-partum (National Health Service, 2019b), expanding the role to cover the needs of mothers returning to work (Royal College of Occupational Therapists, 2024a).

In the UK, ethnic minority women, equating to 14% of the UK population (UK Parliament, 2021), are more likely than those of white British heritage to experience perinatal mental health problems (Royal College of Midwives, 2020). In this article, we use UK ethnicity terminology, which is culturally acceptable and reflects the study protocol and ethical approval. We are aware that this may differ in other countries. In the UK, ethnic minorities is the preferred term defining all ethnic groups except those of white British heritage (Gov.UK, 2021). It also includes some UK-born white minorities such as Gypsy, Roma, and Irish Traveller groups (Gov.UK, 2021). Ethnic minorities also include refugees, from any background and heritage, who are five times more prone to becoming mentally unwell than the general population (UK Parliament, 2023). Despite evidence that a person from an ethnic minority is more likely to experience mental ill-health, they also experience reduced access to mental health services and have poorer health outcomes in the UK (Bansal et al., 2022). Additionally, evidence indicates that systemic racism exists in UK maternity care with poorer outcomes for ethnic minority women (MBRRACE-UK, 2023). A recent scoping study (Sterman and Njelesani, 2021) acknowledged the potential for systemic racism within occupational therapy and called for the profession to be aware of any practices which may inadvertently lead to inequitable outcomes.

## Literature review

The occupational therapy role within perinatal mental health has been recognised in the UK since 2021 (Royal College of Psychiatrists, 2021). Recent evidence has established that the profession clearly articulates its role within the specialism, with occupational therapists undertaking a range of interventions which include lifestyle management, development of positive, maternal co-occupations, and social inclusion activities (Davis and Lovegrove, 2019; Halsall, 2023). Primary research found an occupation-based residential retreat improved mood and self-compassion for a group of women who had experienced pregnancy or infant loss (Hanish, 2019) and that an occupational therapy group intervention helped participants to persevere with breast-feeding, and feel more confident in their parenting role (Sponseller et al., 2021). Evidence from an analysis of occupational therapy practice (Williams and Chard, 2019) indicated that using an evaluation of social interaction supported women to understand the importance of good quality interactions with their babies and social partners during parenting occupations. Occupational therapists have also made effective occupation-based contributions to anxiety management programmes for mothers of premature newborns (Correia et al., 2019)

Whilst Halsall (2023) identified that the occupational therapists in this clinical area are still building an evidence-base to support the efficacy of their interventions, the evidence indicates a clear role for occupational therapists in PCMHS. The Royal College of Occupational Therapists (RCOT) and Health Education England (2020) have created the first online training package to offer practical support to clinicians. Yet, despite a greater recognition of the needs of women within perinatal mental health services in both the UK and internationally, there is little evidence available exploring ethnic minority mothers’ views of occupational therapy.

Edge (2011) found that ethnic minority women in perinatal mental health services were less likely to seek help than white women of British heritage. The study identified higher levels of material, social and emotional deprivation as factors, but only focussed on Caribbean women from one geographical area so findings may not be generalisable. More recently, Amoah (2021) undertook a UK-wide study of 123 ethnic minority women revealing similar data, yet in this study, 81% of the participants were middle to upper class so less likely to be experiencing material and social deprivation.

Ethnic minority women have been shown to have significantly lower contact with PCMHS and be more likely to be detained under the Mental Health Act (Jankovic et al., 2020) suggesting they were in contact with services later in their illness. Jankovic et al. (2020) found that once engaged, ethnic minority women tended to have better attendance than white women of British heritage and suggested the issues were related to access not engagement.

A systematic review (Watson et al., 2019) examined ethnic minority women’s experiences of PCMHS. This study identified that the women had a lack of knowledge of mental health and the available services, alongside a fear of stigma within their community. Service barriers included lack of female staff, language differences, inappropriate interventions, limited cultural knowledge and judgemental attitudes of staff. Practical barriers included travel costs, childcare and appointment times. Ethnic minority women have also reported that the racist approach they perceived from some professionals led to a lack of trust (Edge, 2011; Turienzo et al., 2021).

It has been suggested (Beagan et al. 2022) that interpersonal, institutional and structural racism exists within occupational therapy. There has been some investigation of minoritised people’s experience of broader occupational therapy services. These studies identified unintended but subtle discrimination (Kirsh et al., 2006) and unaddressed language barriers (Kirsh et al., 2006; Mirza and Adare-Harrison, 2018) impacting negatively upon service delivery, service user experience, engagement and outcomes. Yam et al. (2021) suggested that a limited understanding of cultural occupations within the occupational therapist population could also reduce engagement in interventions. Despite calls to increase diversity in healthcare professionals to reduce inequalities (Matthews et al. 2021), registered occupational therapists in the UK are less likely to report as ethnic minority (13% of registrants) compared to other allied health professions (Health and Care Professions Council, 2024).

Whilst systematic review evidence (Crawley, 2022) indicates that occupational therapists have some skills that support their ability to work in a culturally competent manner, perinatal health professions have reported feeling inadequate when working with ethnic minority mothers, particularly asylum seeker mothers due to a lack of cultural awareness training and resources (Edge, 2011; Iturralde et al., 2021). Similar issues are also reported by occupational therapists working in adult and older adult mental health, with clinicians identifying that time developing cultural knowledge, addressing personal biases and finding alternate treatment strategies created work pressures (Pooremamali et al., 2011; Yam et al., 2021).

Perinatal mental health is an established occupational therapy clinical field (Davis and Lovegrove, 2019; Halsall, 2023), yet the evidence identifies barriers for ethnic minority women (Matthews et al., 2021). In the UK, occupational therapists are a key profession in the PCMHS (Royal College of Psychiatrists, 2021); however, their experiences of working with ethnic minority women have not been studied. The aim of this study is to achieve a deeper understanding of the experiences of occupational therapists working in PCMHS. The study, therefore, sought to explore the perceived barriers and enablers to UK occupational therapists in meeting the occupational needs of perinatal mothers from ethnic minority backgrounds.

## Method

The study was approached through a constructivist ontology, recognising the existence of multiple realities and an interpretivist epistemology (Cresswell and Cresswell, 2018) through which we sought to investigate each participant’s unique perception of their practice and the factors which affected this perspective through the reality of their background and experiences. Utilising a qualitative methodology, in-depth interviews enabled an exploration of the perceptions occupational therapists held about their work with mothers from ethnic minorities.

The researchers are all UK-based occupational therapists, each with more than 20 years post-qualification experience. The lead author is a consultant occupational therapist working in perinatal mental health services. This author is committed to ensuring women have equitable access to perinatal mental health occupational therapy. The other authors work in a Higher Education Institution and are dedicated to widening participation in this sector. All are of white British heritage.

Ethical approval for the study was gained from the University of Central Lancashire (HEALTH 0291).

### Recruitment and sampling

A non-probability sampling strategy (Sterba and Foster, 2011), provided a means to explore a small sample from a specific population where resources and time were limited (Field et al., 2006). As an accessible database of perinatal occupational therapists does not exist in the UK, participants were recruited via social media (Twitter, re-named X in 2023). Recruitment through social media has been shown to have value when trying to recruit hard-to-reach populations (Gelinas et al., 2017), and it has been suggested that targeted advertising on specific platforms is more likely to locate participants (Rhodes et al., 2020). The research team were aware that perinatal occupational therapists used the chosen platform and so, to accommodate the study time constraints, this platform was selected for recruitment.

Respondents were invited to participate if they were registered with the UK regulatory body (Health and Care Professions Council); members of their professional body (Royal College of Occupational Therapists); and employed by the NHS and working in an NHS community perinatal mental health service. Participants were excluded if they worked for the lead researcher’s Trust to avoid compromising either party or the findings (Pannucci and Wilkins, 2010).

Occupational therapists who wanted to participate gave consent via an electronic form. Each consenting occupational therapist was invited to participate in a semi-structured interview to share their views.

### Data collection

An interview schedule was developed by one researcher, with questions informed by the literature. A second researcher reviewed the interview schedule. The updated schedule was piloted by two occupational therapists. Further changes were made, as indicated, to produce the final interview schedule.

The final interview schedule (Table 1) consisted of eight introductory questions to collect participant demographics and information about each participant’s employment and previous training. A further eight open-ended questions sought to gain data about participants’ experiences of working with mothers from ethnic minorities, the enablers and barriers that affect treatment, and the support they believed was required to improve their practice.

**Table 1.** Interview schedule.

Demographics	About you	• How would you describe your own ethnic background? • How long have you been registered as an Health and Care Professions Council (HCPC) occupational therapist? • How long have you worked in community perinatal mental health as an occupational therapist?
	About your work	• How much of your clinical time is spent working with mothers from different ethnic backgrounds from your own? • Could you list the different types of ethnic backgrounds the mothers you work with come from?
	Training	• Do you hold any formal post registration training in perinatal mental health? • Can you tell me about any or non-mandatory training, personal development or professional activity in relation to equality and diversity? • How do you feel the training you have received supports your ability to provide occupational therapy to mother from minority ethnic backgrounds?
Experiences	• In your experience what you have found enables your ability to provide occupational therapy to mothers from minority ethnic backgrounds and how have these influenced the occupational therapy you were able to provide? • Can you tell me about any barriers you have encountered when offering occupational therapy these groups of mothers and how do you think these barriers influenced the occupational therapy you were able to provide? • Are you able to tell me if or how you addressed these barriers? • How do you feel your own ethnic background influences the occupational therapy you offer to the mothers you work with? • How have your experiences affected how you feel about working with mothers from different cultural backgrounds and has this changed over time?	
Recommendations	• What do you feel would support you to offer the best occupational therapy service to mothers from different ethnic backgrounds? (Individually? Within your service? Nationally?) • What advice would you give to an occupational therapist starting work in perinatal mental health about working with mothers from minority ethnic backgrounds? • Is there anything else you feel it is important to tell me about your experience of offering occupational therapy to mothers from minority ethnic backgrounds?	

The interviews were conducted by one researcher using Microsoft Teams. Each interview was audio or video recorded depending on the preference of the participant.

### Data analysis

The paucity of perinatal mental health occupational therapy research indicated an inductive analysis to allow theory to emerge through the collection of data rather than draw from existing hypotheses (O’Reilly, 2012). The recording of the interview was auto transcribed verbatim by Microsoft Teams then reviewed for accuracy, and amended as required, by the researcher (KH) who conducted the interviews. A pseudonym was allocated and used throughout the data analysis to protect anonymity. Braun and Clarke’s (2006) Thematic Analysis was used to analyse the data. Data analysis was completed manually. The transcripts were reviewed numerous times to gain a deep familiarity with the data and highlight preliminary ideas. Initial codes were generated identifying elements of the content which presented as significant. This data were systematically studied with any potential themes or patterns relating to the research question noted. These were then reviewed and categorised into overarching and subthemes and the relevant data attached. Themes were then analysed in relation to the research question and given a descriptive title for reporting.

To support a rigorous approach, the researcher used a reflective diary throughout (Jasper, 2005) to identify the researcher’s own beliefs, judgements and practices, and how they may be influencing the research. These were reviewed with a second researcher regularly throughout the study to increase awareness of potential bias and increase trustworthiness of the research (Jasper 2005).

## Results

### The participants

The social media post returned 15 responses. Of these, three occupational therapists did not return consent forms and four declined to partake further citing no ethnic minority women on their caseload, therefore, leaving eight consenting participants. Interviews was between 30–55 minutes.

All participants were female. Seven identified as UK white and one as white mixed background. Four had been registered as occupational therapists for less than 10 years, one between 10 and 20 years and three over 20 years. Six had worked in perinatal mental health less than 5 years and the other two between 5 and 10 years.

The participants estimated the percentage of clinical time spent with women from ethnic minorities. This varied from 3% to 60%. Three therapists perceived that their caseload was representative of the local population, but two believed it was disproportionate and unreflective of the potential morbidity of ethnic minority groups. All participants had completed mandatory equality and diversity training through their employers with three undertaking enhanced training.

### Thematic analysis

There were three overarching themes each with subthemes. (1) Observation of caseload (subthemes: concern about caseload demographics, experience of cultural barriers facing women). (2) The experience of providing occupational therapy (subthemes: finding a language for occupational therapy, being person-centred, standardised assessments). (3) The influence of therapist’s own culture (subthemes: awareness of impact of own ethnicity, the importance of cultural awareness).

#### Theme 1: Observation of caseload

All participants discussed their caseload and offered observations of the number of ethnic minority women. They also identified cultural barriers they believed reduced the accessibility of the occupational therapy services.

##### Concern about caseload demographics

Participants expressed concern about the demographics of their caseload with six describing not having many ethnic minority women. They spoke of their sense of responsibility to find a solution to improve access for ethnic minority women. One summarised this concern:

I do in general feel we’re not reaching the women who need us and that bothers me as we haven’t done very well in reaching out to women who reflect our local populations. It makes me feel like I have a kind of responsibility to find a solution (Adie)

Two participants with representative caseload numbers were still not confident that there were adequate numbers of ethnic minority women for a correct balance, suspecting morbidity was unrepresented. One spoke about a recent audit:

So, we did a bit of work recently to check if people were able to access us. I think that’s all well, but it didn’t allow for any difference in terms of prevalence of mental health issues in those populations. I have heard that migrants actually experience higher mental health issues and that doesn’t reflect in our demographics. (Kay)

Another participant expressed concern that colleagues did not refer ethnic minority women to occupational therapy:

I think my team have a lot of women from different ethnic backgrounds, but I don’t feel like many get referred to myself, I don’t know why. (May)

##### Experience of cultural barriers facing women

All participants reported that some ethnic minority women they worked with perceived mental health stigma from their own families or communities, and that sometimes interventions created embarrassment:

We have offered OT [occupational therapy] but sometimes it’s declined, and that’s really difficult because they didn’t want to be seen out with us. They didn’t want to raise awareness; they were worried that their community would make assumptions or wouldn’t be accepting. (May)

Four participants reported that women were afraid of engaging with services, due to perceived consequences including removal of their children, being dismissed or experiencing racism. Personal trauma or shame of mental illness also created barriers. Participants identified particular challenges of home-based occupational therapy interventions and of being potentially unwelcome guests. One described her experience of working with Irish Travellers:

I think sometimes we’re viewed as one of the services not there to support but to judge, I think I’ve felt that quite a lot. We’re also interested in the environment where someone lives. In the perinatal period most occupations take place in the home. For example, for an Irish Traveller having someone in their space is really uncomfortable and that has been quite challenging. (Adie)

Five participants reported that a combination of cultural views of motherhood, mental health and knowledge of occupational therapy could create misunderstandings affecting both mothers and therapists. They conveyed working with different values, expectations of women and cultural nuances required sensitivity. All voiced the importance of working with the whole family unit and investing extra time to improve initial engagement.

I was working with a South Asian lady who really wanted to be in a mothering role but was struggling to get out of a sick role. It was all or nothing. I was setting small goals but had to explain her limits to the whole family and that just because she could cook one meal, she couldn’t do everything. When I took time to explain it, they understood. It really helped. Before that she had been stuck not only by her own expectations but by her family’s. (Esme)

#### Theme 2: Experience of providing occupational therapy

##### Finding a language for occupational therapy

Language was a barrier for all participants. They reported that mental health did not translate clearly, and written information was inadequate. Explaining occupational therapy in the context of other cultures was particularly complicated, as one participant stated:

I think trying to explain what OT [occupational therapy] is, is really difficult, particularly if she hasn’t encountered OT before. Trying to explain the unique thing you bring and how it will fit into her culture, her situation, is hard. (Sue)

The experience of using interpreters was mixed. Four described positive instances finding specialised or regular interpreters helpful:

He [the interpreter] was fantastic, he totally got the tension coming from a culture that says, ‘I do what I’m told’ and saying to the woman you have a voice – what is it what you want to be able to do? (Jane)

However, five participants reported negative experiences indicating interpreters were not always accurate, altered communication flow, created confusion and reduced trust:

Quite often people don’t want to talk in front of an interpreter, they are worried the interpreter will judge them so they’re not honest. There’s just too many people, information gets lost in translation and it’s so much harder for that woman to talk about how she’s feeling. (Lucy)

##### Importance of being person-centred

All participants believed individualised care enabled women to engage with occupational therapy believing it was achieved through the core values of the profession:

What helps is OTs’ [occupational therapists’] recognition of people as individuals and what they think is important to them, what they want and how they want to live their life. Recognising and celebrating differences, enabling people to do things in their own way and how they want to do. (Kay)

Three participants felt that recognising the universal similarities of motherhood was as important as acknowledging cultural differences. One articulated:

I think I try to treat everyone as a human being, as a mum who has her own worries, concerns and anxieties, and difficulties around function as much as any other mum who’s starting on this lifelong journey of motherhood. (Jane)

##### The challenges of standardised assessments

All participants reported that it took extra time to achieve a comprehensive assessment with women of an ethnic minority. They expressed concern about an insufficient range of culturally appropriate standardised formats and admitted adapting them to meet needs. One participant spoke of her experience of using an assessment:

The OSA [Occupational Self-Assessment] is brilliant for white middle class women, but I learned I had to be much more flexible with ethnic minority women, using a more conversational style to draw out what was relevant, then linking it to broad OT [occupational therapy] themes for my notes – yeah letting go of traditional tools was helpful. (Adie)

#### Theme 3: Influence of therapist’s culture

##### Awareness of impact of own ethnicity

The occupational therapists indicated that they believed their ethnicity influenced their practice. Two spoke of it preventing engagement, one gave an example of her experience:

I’m not from those backgrounds and I’ve had women sort of say to me you’re really nice, but you just don’t understand what it’s like for women like me. (Esme)

Two participants, however, found being UK white assisted, with one participant reporting:

I think sometimes I have found particular cultures are really keen to talk to me because I’m a white woman. They say within their own culture talking about these things would be frowned upon and they would be thought of as mad or bad. (Gill)

Participants reflected how unconscious bias may affect the accuracy of their assessments and the appropriateness of their interventions. One candidly disclosed:

There’s probably unconscious biases there and things that we assume. I think our own identity, upbringing and experiences naturally impact upon our practice. For example, I might just assume something like someone’s parenting or doing an activity is wrong and it just might be really different in their culture. I’m sure there’s stuff I’ve assumed, perhaps wrongly so. I think that’s something that impacts upon my practice. (May)

Six participants reported using reflexive practice to challenge their biases with three believing it should be part of occupational therapy training, one advised:

For OT [occupational therapy] it’s about always reflecting on yourself and not being surprised if something is different. Being open to who the person is and what’s important to them rather than ladling them with like, OK you live on the 18th floor, and you can’t do this because you’re Bangladeshi. Just making sure you don’t just put all those connotations upon someone, and you make sure you see them and think about what it is they want within that. (Gill)

##### Importance of cultural awareness

Seven participants worried that having limited cultural knowledge impacted negatively upon their practice. Fears included missing essential information, inadvertently causing offence or making poor clinical choices:

I think I feel not properly skilled. I worry about doing the right thing or saying the wrong thing. I think that my lack of confidence, not wanting to get it wrong can consume me at times. It affects how I practise, and I think the person gets different, not, not good care but I think it’s a bit of a hang up. (Sue)

All eight occupational therapists expressed the frustration of not being able to attain a knowledge of all cultures. Two described how the fusion of second- and third-generation cultures created further distinctions. They all reported how limited knowledge hindered understanding. One occupational therapist reflected:

There are lots of cultural beliefs about being pregnant, having a baby, the role of the wider family network and I don’t think I always appreciate or understand when it’s different from the known. For example, the stereotype of South Asian families being really helpful, that’s not always the case with second generation women who feel like they fit between two cultures. They don’t always want or get offered what I’d expect. I don’t know how well equipped we are if I’m honest, but we’re sensible, we try very hard. (Adie)

Participants described their strategies. They spoke of prioritising time to understand the woman within her culture, being curious, seeking clarification, and acknowledging ignorance as important to achieve engagement and good assessments:

I say look I’m not from the same place so I’ve got no idea what this means for you, but I think that can help with the collaborative process, I’m an OT [occupational therapist], that’s how I work. I’m not presenting myself with all the answers, I love how it empowers the mums. (Jane)

Participants’ cultural confidence appeared to increase with experience. All found seeking advice from colleagues of different ethnicities helpful and reported that a more diverse occupational therapy workforce could improve team practice. All indicated that they believed ethnic minority peer support workers and community engagement may also be beneficial. One participant identified utilising their local equality and diversity team. All thought better training was required, with seven indicating that the current mandatory training provision was inadequate and five believing that the occupational therapy professional body (RCOT) should make better provision. Participants suggested cultural training should be more reflective, occupationally focussed and applied to the perinatal period:

It’s hard because you can’t train in or learn every culture but equally, learning a little about each with more of an OT [occupational therapy] spin on it and think about the links to motherhood would be good. (Sue)

## Discussion

This study sought to achieve a deeper understanding of the experiences of occupational therapists working in PCMHS. It has explored the perceived barriers and enablers to UK occupational therapists in meeting the occupational needs of perinatal mothers from ethnic minority backgrounds.

Occupational therapists in this study believed that their services were not fully accessible to all women with participants perceiving that their caseloads were not reflective of the perinatal ethnic minority population they served. This is congruent with a recent study (Jankovic et al. 2020) of UK PCMHS referrals in which the ethnic minority population was proportionately under-represented across the service. There are several possible reasons for this. Stigma, fear, language, lack of cultural understanding of mental health and the expectations of motherhood have been identified as barriers to ethnic minority mothers accessing and engaging with a PCMHS (Iturralde et al., 2021; Jankovic et al. 2020; Watson et al. 2019). Whilst these barriers were indicated in our study, other barriers such as inconvenient appointment times, childcare and travel costs (Iturralde et al., 2021; Jankovic et al. 2020; Watson et al., 2019) were not. We suggest that the flexible nature of where the occupational therapists offer their interventions may alleviate some of these barriers.

Occupational therapists participating in this study did suspect their own ethnicity created an unconscious bias which affected their practice, potentially leading to ethnic minority women disengaging from the service and impacting on the delivery of occupational therapy interventions. These findings are supported by Edge (2011) and Turienzo et al., (2021) who identified that ethnic minority women had experienced bias and prejudice when encountering perinatal services. When viewed in this context it is unsurprising that building trust may be more complex in subsequent health interactions. Cross-cultural issues in occupational therapy have also been identified by Yam et al. (2021) who recommend occupational therapists integrate cultural humility into their practice. The participants in our study supported this proposition, identifying a holistic, person-centred approach and the use of the universal language of motherhood as key to engagement.

Our study proposes that a further possible reason for the under-representation of ethnic minority mothers accessing PCMHS, was healthcare colleagues not identifying ethnic minority mothers’ need for occupational therapy; therefore, referrals into the service were being overlooked. Whilst this is an original finding for perinatal community mental health, similar issues have been identified in adult community mental health (Parkinson et al., 2009) suggesting this may be reflective of a broader issue within the profession. Wilding (2010) stated many occupational therapists report difficulty articulating their profession, with Parkinson et al. (2009) recommending the use of occupational therapy models to help occupational therapists explain their role. Whilst this approach could be helpful for communication with other professionals, this is likely to be more complicated where differences in cultural perceptions and language to describe mental illness and other aspects of healthcare exist between the occupational therapists and the people with whom they work (Watson et al., 2019). Further, occupational therapists should take every opportunity, utilising occupational therapy models, where appropriate to educate other professions about the role of the profession and the importance of referring ethnic minority women who may benefit from the service.

Our study supports findings by Matthews et al. (2021) that greater diversity within the occupational therapy workforce and the PCMHS would improve cultural competency enabling more culturally sensitive care. This may remove or moderate some of the barriers described in our findings. The need for diversity and inclusion is recognised by occupational therapy bodies internationally (Royal College of Occupational Therapists, 2023; World Federation of Occupational Therapists, 2020). Current Royal College of Occupational Therapists strategies aim to enable those from historically marginalised and under-represented communities to enter and progress within the profession (Royal College of Occupational Therapists, 2023, 2024b). At present only 8% of the current UK occupational therapy workforce are from an ethic minority background (NHS Digital, 2018) and occupational therapists and students from this proportion of the workforce have indicated that they feel of lack of belonging in their professional context. Alongside professional bodies, employers, regulators and higher education institutions (Atwal et al. 2021), occupational therapists need to take active steps to increase awareness of current recommendations and to enact these to effect change. This includes listening, taking action and speaking out (Atwal et al. 2021) and utilising allyship strategies (Lutra, 2022) to promote diversity and inclusivity.

This study suggests that individual occupational therapy practitioners do not feel culturally competent. Whilst the participants indicated an enthusiasm for gaining cultural understanding, they did not feel mandatory training equipped them with the knowledge to meet the needs of ethnic minority mothers. Occupational therapists need to highlight this to managers, and organisations need to take responsibility to address this issue. In addition, occupational therapists should challenge their cultural assumptions using reflexive journals, supervision, and the advice of ethnic minority colleagues to develop greater cultural awareness. These techniques, supported by our findings and those of others (Sterman and Njelesani, 2021), should be promoted to staff in PCMHS.

Participants raised concerns that culturally inappropriate standardised occupational therapy assessments resulted in poor treatment outcomes for those ethnic minority women. This is an original finding for occupational therapy in PCMHS and is supported by similar concerns in other mental health settings (Pooremamali et al., 2011; Yam et al., 2021). Participants in our study strived to adapt their practice to make the assessments more relevant. This required additional time and consequently increased work pressure. It also put the validity of the standardised assessment at risk due to practitioners altering formats to meet the needs of the service users. If occupational therapists are to reduce these barriers, they need to collaborate with manufacturers and developers and demand greater cultural sensitivity in standardised assessments.

### Strengths and limitations

The ethnic representation of the participants was approximately the same as the UK occupational therapy workforce (NHS Digital, 2018), supporting the use of X as a recruitment strategy for this study. Whilst only one researcher collected and analysed the data, a reflexive approach and regular discussions with a more experienced researcher offered some mitigation of this approach. The researchers were UK-based occupational therapists of white British heritage which potentially created observation bias during the study. This was also considered through reflexive processes and supervision. Whilst the themes identified demonstrated there were clear patterns emerging in the data collected, it was unclear if data saturation was achieved, and a longer recruitment period from a wider range of social media platforms would have either confirmed saturation or facilitated the recruitment of additional participants bringing new concepts. Although this is a limitation in this study, the authors have confidence that the depth of the data collected provides a comprehensive picture of the current experiences of occupational therapists working in PCMHS.

Whilst the focus of our work relates to people who may be marginalised by healthcare services, this study aimed to explore the barriers and enablers perceived by occupational therapists in responding to the occupational needs of perinatal mothers from ethnic minority backgrounds. We recognise that a feminist epistemology would be suited to qualitative exploration of the needs of people experiencing marginalisation and power imbalance (McHugh, 2014). This approach would have provided an alternative lens to guide the collection and analysis of the data, and this may have led to a different interpretation. Whilst the methodology underpinning our study was aligned with the research aim and the context to the study, we do recognise that this may have limited the work and we recommend exploration of the experiences of perinatal ethnic minority mothers.

### Implications

Occupational therapists in PCMHS perceive that barriers exist to prevent ethnic minority mothers fully accessing and benefitting from their services. Although the study was undertaken in a UK setting, the recommendations may be of value to occupational therapists working with women in other global settings. Ultimately, this study identifies the need to increase the diversity of the occupational therapy workforce. To achieve this goal, occupational therapists need to collaborate with the people with whom they work, professional bodies and other stakeholders to increase the number of ethnic minority occupational therapy students and practitioners.

Our findings also suggest that occupational therapists should strive to further develop greater cultural sensitivity in their practice. Possible strategies to enable more responsive services may include consideration of the setting in which interventions are offered and access to interpreters experienced in the specific clinical field, in this case PCMHS, to facilitate interactions. Occupational therapists working in PCMHS should also consider how they can build a more reflexive approach to their practice to increase awareness of unintentional prejudice and enable inclusivity. A range of techniques are available to support this, including the use of reflexive diaries, reflective supervision, and tools such as the RCOT 5-minute reflection (Royal College of Occupational Therapists, 2020). Mandatory training should be designed to better prepare PCMHS staff to provide culturally appropriate and individualised interventions. Employers need to recognise and seek to understand clinicians’ concerns about poor cultural awareness, its impact upon service delivery, and undertake the actions necessary to ensure training is fit for purpose. Occupational therapists need to take responsibility and action to ensure that organisations and developers of occupational therapy assessments provide resources that support inclusion in the PCMHS. Further research exploring the views of ethnic minority mothers will provide a more comprehensive picture and guide future recommendations.

## Conclusion

Through exploration of the perceptions of occupational therapists working in PCMHS, this study identified a number of factors which affected the provision of occupational therapy to women from ethnic minorities. Barriers to service access for ethnic minority women included stigma, fear, language, lack of cultural understanding of mental health and the expectations of motherhood and colleagues not acknowledging the need for occupational therapy. Within occupational therapy services, factors affecting the quality and effectiveness of interventions included a lack of culturally appropriate occupational therapy assessments and the therapist’s own ethnic background. There is a clear need to improve the diversity within the occupational therapy workforce in PCMHS as well as more widely. Whilst current mandatory training methods did not improve cultural awareness, cultural humility and reflexive practice were perceived to support the delivery of more effective practice. Recommendations from this study indicate that occupational therapy needs to gain a deeper understanding of the barriers facing ethnic minority mothers and their therapists. Actions to improve diversity and inclusion are a priority for all occupational therapists and are essential if PCMHS occupational therapists are to provide an accessible and high-quality service for mothers of all ethnicities.

**Key findings**

Occupational therapist participants identified that

- Ethnic minority women experience barriers to accessing occupational therapy perinatal mental health services
- Current mandatory training methods did not improve cultural awareness
- Greater diversity in the occupational therapy workforce would improve access to their services

**What this study has added**

This study increases understanding of the perceived barriers and enablers reported by UK occupational therapists when responding to the occupational needs of women from ethnic minority backgrounds experiencing perinatal mental health illness. Based on these barriers, a range of strategies to improve cultural competence and relevance in perinatal mental health occupational therapy services are proposed.
